# Supplementary material for: Clinical Outcomes of the Intraocular Lens Injector and Busin Glide for Descemet Stripping Automated Endothelial Keratoplasty in Patients with Iridocorneal Endothelial Syndrome
Source: J Clin Med. 2023 Feb 26;12(5):1856. doi: 10.3390/jcm12051856 (PMC10003726; doi:10.3390/jcm12051856)
Supplement: Supplementary file 1 [file jcm-12-01856-s001.zip › jcm-2177215-supplementary Figrues S1-S3.pdf]

Supplemental Figures

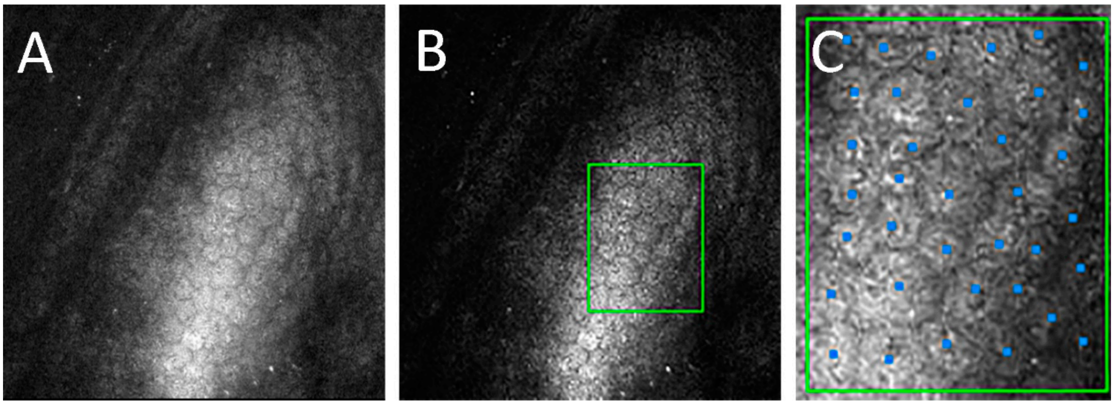

Supplemental Figure S1. The method of endothelial cell counting. (A) A clear image of endothelium. (B) Using the green frame to select the local area in the image manually. (C) All cells inside the frame were identified by using a point-and-click method.

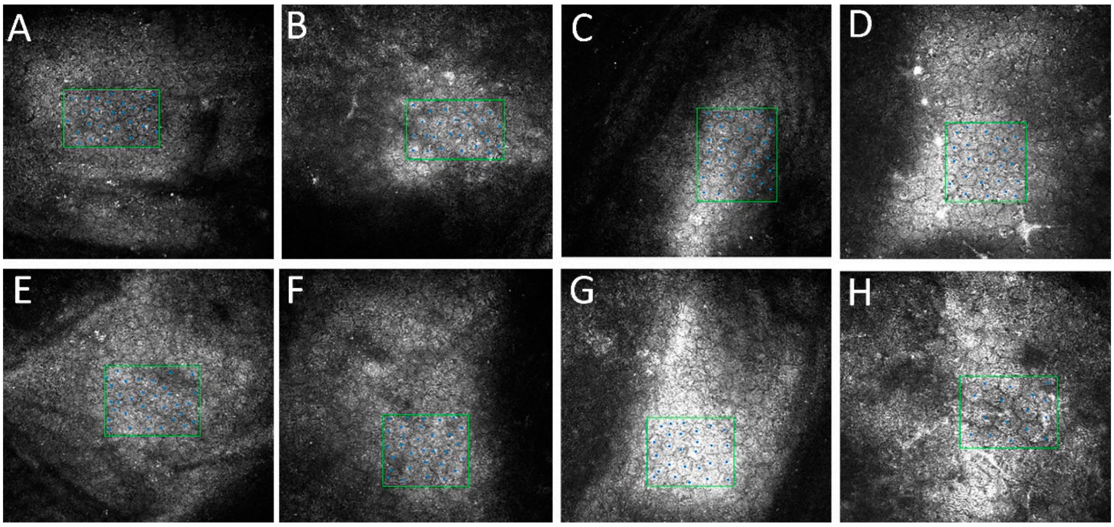

Supplemental Figure S2. Endothelial images recorded by confocal microscope of the two patients who underwent DSAEK with different graft insertion techniques. (A~D) A representative patient who underwent DSEAK using the injector. A to D respectively showed the endothelial cell density and morphology of the patient at 1 month, 3 months, 6 months and 1 year after operation. (E~H) A representative patient who underwent DSEAK using the Busin glide. E to H respectively showed the endothelial cell density and morphology of the patient at 1 month, 3 months, 6 months and 1 year after operation.

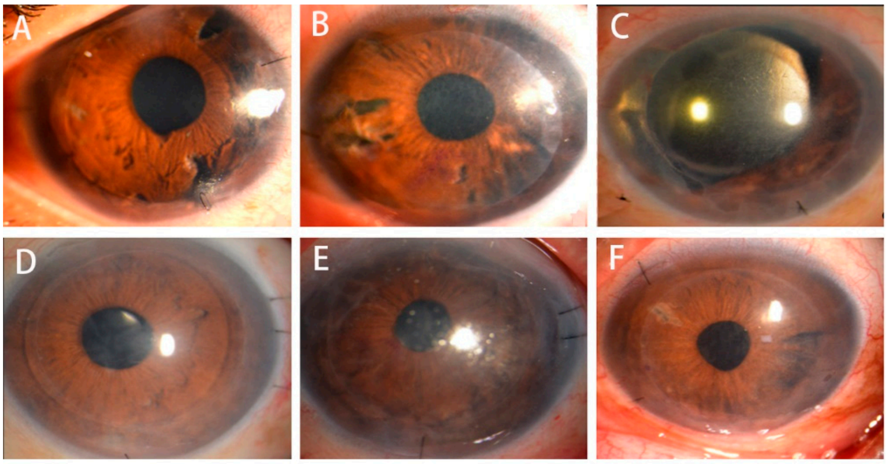

Supplemental Figure S3. Different degrees of iris damage in eyes with ICE syndrome after DSAEK. (A-C) Relatively serious iris damage of eyes in the Busin group. (D-F) Relatively slight iris damage of eyes in the injector group.
